# *In Vitro* Acylation of Okadaic Acid in the Presence of Various Bivalves’ Extracts

**DOI:** 10.3390/md11020300

**Published:** 2013-01-29

**Authors:** Keiichi Konoki, Tatsuya Onoda, Ryuichi Watanabe, Yuko Cho, Shinnosuke Kaga, Toshiyuki Suzuki, Mari Yotsu-Yamashita

**Affiliations:** 1 Graduate School of Agricultural Science, Tohoku University, 1-1 Tsutsumidori-Amamiyamachi, Aoba-ku, Sendai 981-8555, Japan; E-Mails: b1am1310@s.tohoku.ac.jp (T.O.); choyuko@m.tohoku.ac.jp (Y.C); myama@biochem.tohoku.ac.jp (M.Y.-Y.); 2 National Research Institute of Fisheries Science, 2-12-4 Fukuura, Kanazawa, Yokohama 236-8648, Japan; E-Mails: rwatanabe@affrc.go.jp (R.W.); tsuzuki@affrc.go.jp (T.S.); 3 Iwate Fisheries Technology Center, Kamaishi 026-0001, Japan; E-Mail: s-kaga@pref.iwate.jp

**Keywords:** okadaic acid, dinophysistoxin, *Halichondria okadai*, okadaic acid binding protein, diarrhetic shellfish poisoning, acyl coenzyme A transferase, detoxification

## Abstract

The dinoflagellate *Dinophysis* spp. is responsible for diarrhetic shellfish poisoning (DSP). In the bivalves exposed to the toxic bloom of the dinoflagellate, dinophysistoxin 3 (DTX3), the 7-OH acylated form of either okadaic acid (OA) or DTX1, is produced. We demonstrated *in vitro* acylation of OA with palmitoyl CoA in the presence of protein extract from the digestive gland, but not other tissues of the bivalve *Mizuhopecten yessoensis*. The yield of 7-*O*-palmitoyl OA reached its maximum within 2 h, was the highest at 37 °C followed by 28 °C, 16 °C and 4 °C and was the highest at pH 8 in comparison with the yields at pH 6 and pH 4. The transformation also proceeded when the protein extract was prepared from the bivalves *Corbicula japonica* and *Crassostrea gigas*. The OA binding protein OABP2 identified in the sponge *Halichondria okadai* was not detected in the bivalve *M. yessoensis*, the bivalve *Mytilus galloprovincialis* and the ascidian *Halocynthia roretzi*, though they are known to accumulate diarrhetic shellfish poisoning toxins. Since DTX3 does not bind to protein phosphatases 1 and 2A, the physiological target for OA and DTXs in mammalian cells, the acylation of DSP toxins would be related to a detoxification mechanism for the bivalve species.

## 1. Introduction

**Figure 1:**
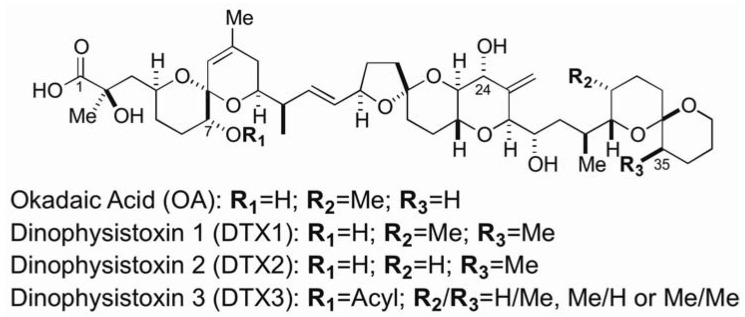
Structures of okadaic acid (OA), dinophysistoxin 1 (DTX1), dinophysistoxin 2 (DTX2) and dinophysistoxin 3 (DTX3).

Diarrhetic shellfish poisoning (DSP) was first recognized in Japan along the coast of the Tohoku Area in the 1970s [1], where the dinoflagellate *Dinophysis fortii* was dominantly identified in the toxic bloom [2]. Okadaic acid (OA) and dinophysistoxins (DTXs) were isolated from the digestive glands of shellfish [3,4] and were deduced to be DSP toxins (Figure 1). The structure of OA, DTX1 and DTX2 were determined in 1981 [5], 1982 [3] and 1992 [6], respectively. Since DSP causes gastrointestinal dysfunction, which accompanies diarrhea, vomiting and abdominal pains, the local governments periodically monitor the appearance of *Dinophysis* spp. and quantitate toxin concentration in shellfish in order to determine whether to suspend shipments during the period when the toxin level was beyond 0.05 mouse unit/g. DTX3, named for 7-*O*-acylated derivatives of OA or DTX1, was not synthesized by the dinoflagellate, in spite of being present in bivalves [7]. The ratio of acylated forms to non-acylated forms detected in digestive glands of DSP-exposed (toxic) bivalves depended on the bivalve species. It was reported that Japanese scallops possess a higher capability to convert DTX1 to DTX3 in comparison with mussels [8]. *In vitro* transformation reaction reported by two research groups [9,10] suggested that bivalves are considered as the platform for the transformation reaction, ruling out the bacterial contribution [11] in this transformation. 

Shellfish poisoning used to be classified in four groups depending on the symptoms; Diarrhetic shellfish poisoning (DSP), paralytic shellfish poisoning (PSP) [12], neurotoxic shellfish poisoning (NSP) [13] and amnesic shellfish poisoning (ASP) [14]. Now, the classification is based on the chemical characteristics of shellfish poisoning toxins groups [15]. The eight groups are: the azaspiracid group, brevetoxin group, cyclic imine group, domoic acid group, okadaic acid group, pectenotoxin group, saxitoxin group and yessotoxin group. For all these groups, the producers and structures of responsible toxins have already been revealed. The transformation of some of these toxins occurring in bivalves as observed in DSP toxin-carrying shellfish has not been investigated for all the toxins. In bivalves that accumulate PSP toxins, multiple products with various levels of toxicity were produced after the transformation reaction [16]. For DSP toxins, besides the acylation at C7-OH as described earlier, the diol esters of OA [17] were hydrolyzed to OA [18]. In addition, DTX4 [19] and DTX5 [20], produced by the dinoflagellate *Prorocentrum* spp., have not been reported in bivalves to date. Hydrolysis of diol esters of OA or DTXs 4 and 5 leads to an increase of toxicity by restoring binding ability to serine/threonine protein phosphatases 1 (PP1) and 2A (PP2A), the primary target for OA in mammalian tissues [21]. Since DTX3 lacks binding ability to PP1 and PP2A [22], acylation would decrease fatal risk to the animals accumulating the DSP toxins. An intriguing observation, however, was made by Yanagi *et al*. that DTX3 retained its ability to cause diarrhea and exhibit cytotoxicity [23]. Therefore, the physiological significance of acylation of DSP toxins to bivalve species was not clearly understood until now.

OA was originally isolated from the marine sponge *Halichondria okadai* [5]. OA specifically bound to PP1 and PP2A with 150 nM and 30 pM of dissociation constant [22], which led to an exhibition of tumor promotion [24]. Since then, OA has been widely used for basic research. X-ray crystal structure revealed the well-folded structure of OA in the catalytic subunit of PP2A, showing the presence of an intramolecular hydrogen bond between the carboxyl group and C24-OH [5,25]. PP1 and PP2A are two of the fundamental proteins in all the living species, and their sequences are highly conserved. OA binding proteins OABP1 and OABP2 were isolated from *H. okadai* [26]. The amino acid sequence of OABP1, partially deduced from the cDNA sequence, was 88% homologous to rabbit PP2A. On the other hand, OABP2 was not homologous to any protein phosphatases reported in the literature. Since a homology search did not let us infer the function of OABP2, curiosity was raised on the physiological roles of this protein. OABP2 was composed of three homologues: OABP2.1, 2.2 and 2.3. OABP2.1 was 98% and 66% homologous to OABP2.2 and OABP2.3, respectively. Among them, OABP2.1 and 2.3 were stably expressed as recombinant proteins in *E. coli* [27]. Since OA bound to the recombinant OABP2.1 and 2.3 with 1.30 ± 0.56 nM and 1.54 ± 0.35 nM of dissociation constant, the binding site of OA would be located in sequences that OABP2.1 and 2.3 have in common. OABP2 was not identified in crude extracts of the sponge *H. japonica*, which is often seen in the same habitat as that of *H. okadai*. Thus, the presence of OABP2 seemed to be physiologically important to *H. okadai*, though the presence of OABP2 in toxic bivalves has not been investigated so far.

We herein investigated toxic and non-toxic bivalves by Western blotting using an anti-OABP2 serum in order to assess the expression of the protein in the bivalve species. Next, we compared *in vitro* transformation of OA into DTX3 in the presence of crude extracts from different tissues of the scallop *Mizuhopecten yessoensis* to identify the location of the transformation reaction. We also tested two other bivalve species, *Corbicula japonica* and *Crassostrea gigas*, to determine whether they were able to transform OA into DTX3 and to address the generality of the transforming ability.

## 2. Results and Discussion

### 2.1. Okadaic Acid Binding Proteins in Shellfish

**Figure 2:**
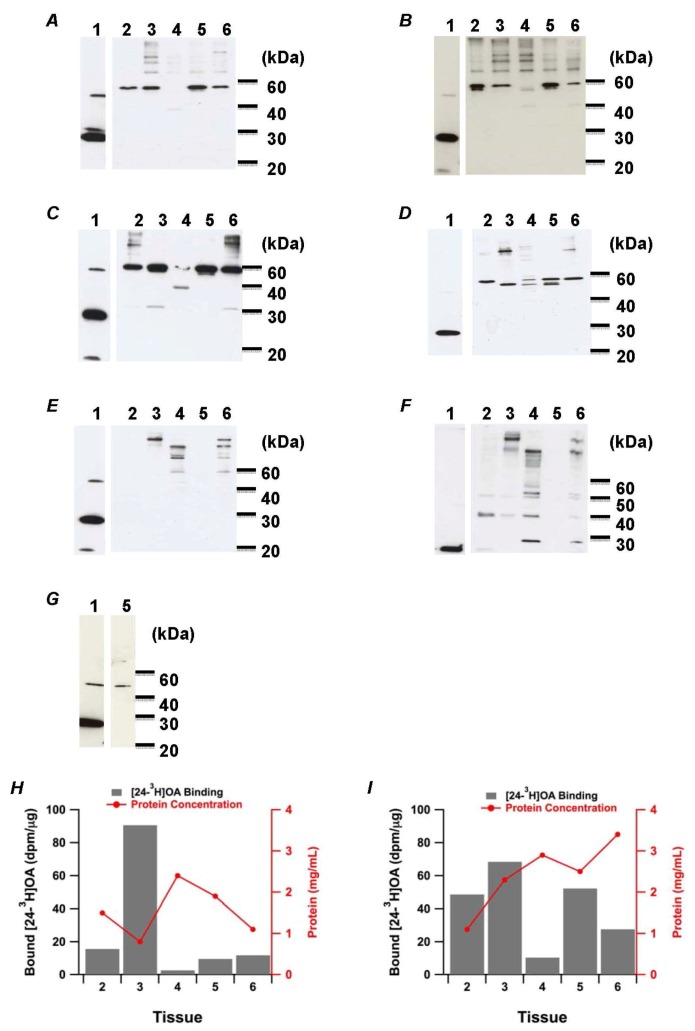
Western blotting analysis of shellfish extracts with anti-OABP2.1 serum. (**A**–**E**) Extracts were prepared from the nontoxic scallop *Mizuhopecten yessoensis* (**A**, **B**), the toxic scallop *M. yessoensis* (**C**, **D**), the toxic *Mytilus galloprovincialis* (**E**, **F**) and toxic ascidian *Halocynthia roretzi* (**G**). The pellet fractions were A, C and E. The supernatant fractions were B, D and F. The whole homogenate was used for *H. roretzi*. Lanes 1–6 correspond to recombinant OABP2.1, gills, gonad, adductor muscle, digestive gland and mantle, respectively. (**H**, **I**) Binding of [24-^3^H]OA to the pellet fraction (**H**) and supernatant fraction (**I**) of nontoxic *M. yessoensis* (grey bars). Protein concentration of each fraction was represented with red lines. Lane description was the same as described for A–G.

Western blotting analysis of the crude extracts was carried out to identify the OA binding protein OABP2.1 in the nontoxic and toxic shellfish extracts. From the scallop *Mizuhopecten yessoensis* and the mussel *Mytilus galloprovincialis*, the gills, gonad, adductor muscle, digestive gland and mantle were subjected to Western blotting. Since it was difficult to separate each tissue from the ascidian *H. roretzi*, only the digestive gland was used for preparing the extract. The results indicated that none of the extract from non-toxic *M. yessoensis* (Figure 2A,B), toxic *M. yessoensis* (Figure 2C,D), *M. galloprovincialis* (Figure 2E,F) and the ascidian *H. roretzi* (Figure 2G) appeared to contain OABP2.1, whereas the antiserum detected an intense amount of recombinant OABP2.1 around 22 kDa (lane 1). In fact, the anti-OABP2.1 serum detected some different-sized proteins. The antiserum primarily detected *ca.* 60 kDa-sized proteins in every tissue, except the adductor muscle from nontoxic and toxic *M. yessoensis* (lanes 2, 3, 5 and 6, Figure 2A–D). On the other hand, the antiserum did not exhibit distinctive labeling in the digestive gland from *M. galloprovincialis* (Figure 2E,F) and *H. roretzi* (Figure 2G), while it labeled multiple proteins in the adductor muscle (lane 4) and mantle (lane 6) from *M. galloprovincialis* (Figure 2E,F).

OA binding proteins were searched for in each tissue extract of nontoxic *M. yessoensis* by examining [24-^3^H]OA binding (Figure 2H,I). In the results, the pellet fraction from the gonad (lane 3, Figure 2H) showed the highest binding of the radioligand, and the supernatant fraction from the gills (lane 2), digestive gland (lane 5) and mantle (lane 6) showed moderate binding. Both of the pellet and supernatant fractions from the adductor muscle (lane 4) had relatively weak binding. 

Phosphorylation and dephosphorylation are the fundamental signal transduction processes and are targeted by many natural toxins [28,29]. OA specifically inhibits protein phosphatases 1 and 2A with 150 nM and 30 pM of the dissociation constant, respectively [22], which leads to the appearance of cytotoxicity [30,31] and cancer promotion [24,32]. Secondary metabolites, such as OA, isolated from marine sponges, are not considered as sponge metabolites, but as metabolites of a symbiotic species [33,34]. Due to difficulty in the purification of such symbiotic species from the sponges, research studies have been aimed towards the cloning of secondary metabolite-producing genes from metagenome [35]. However, the metagenomic approach has not been successful with all the targeted compounds. In order to purify the secondary metabolite-producing species, it would be important to understand the symbiotic relationship by identifying the clue substance(s) that affect the host species, as well as the symbiotic species. 

OABP2, the OA binding protein isolated from the sponge *H. okadai*, was postulated as such a key substance that would regulate the concentration of OA in the sponge to reduce self-toxicity and unleash OA to exhibit cytotoxicity when undesired species come into the sponge. We hypothesized that other OA-accumulating species might possess OABP2. In this study, however, OABP2 was not identified in bivalves that accumulate DSP toxins when the toxic bloom occurs (Figure 2). The anti-OABP2 serum almost equally labeled a protein around 60 kDa in each tissue extract from *M. yessoensis*, while it labeled different sized protein(s) from extracts prepared from other species. It was unlikely that the proteins detected by anti-OABP2.1 serum in each tissue were the OA binding proteins, since the [24-^3^H]OA binding profile did not support the Western blotting results. The results comprised the presence of multiple binding proteins for OA in the *M. yessoensis*, which was compatible with the observation made by Rossignoli *et al*., where various sized proteins were bound to OA more or less nonspecifically [36].

### 2.2. *In Vitro* Transformation of OA into 7-*O*-Palmitoyl OA (DTX3) in *M. yessoensis*

*In vitro* transformation reaction of OA was performed in the microsomal fraction and the mitochondrial fraction prepared from the digestive gland of nontoxic *M. yessoensis*. Both of the fractions turned out to show OA-transforming ability as described later, which was compatible with the observation by Rossignoli *et al.* [10]. We then decided to investigate the transformation reaction in the microsomal fraction [37]. After 2 h of the reaction, quantitation of unreacted OA and 7-*O*-palmitoyl OA in the AcOEt layer was carried out with LC-MS/MS with [M − H]^−^ as the precursor ion and *m/z* 255 as the product ion [38]. The multiple reaction monitoring (MRM) peak with *m/z* 803 > 255 (Q1 > Q3) detected at 10.6 min was assigned to that of unreacted OA. Under this condition, however, the MRM peak with *m/z* 1041 > 255, corresponding to that for 7-*O*-palmitoyl OA, was not detected. 

**Figure 3:**
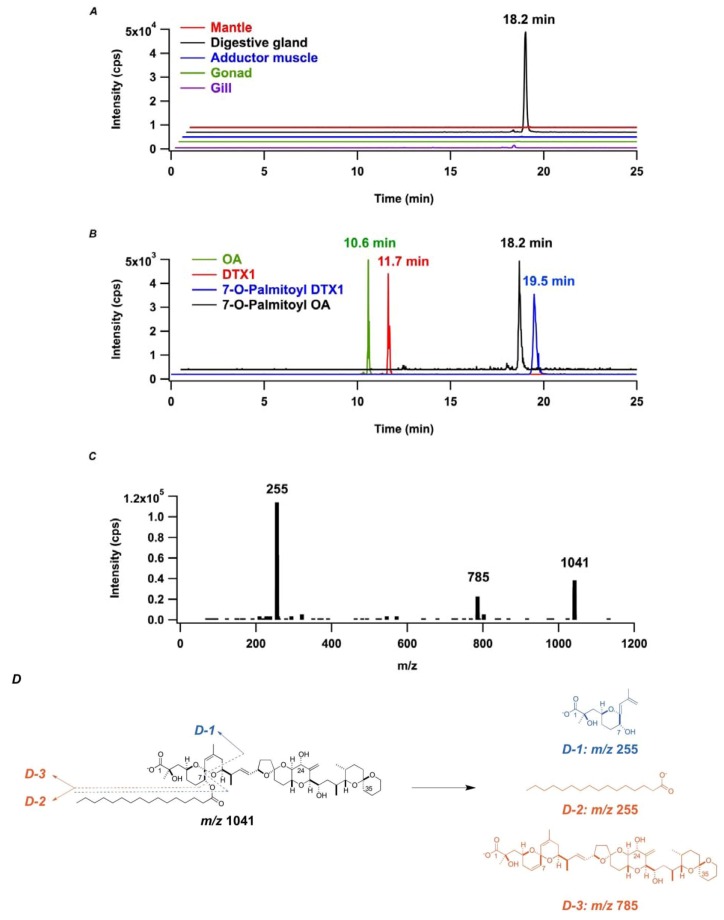
Identification of 7-*O*-palmitoyl OA in *in vitro* transformation reaction mixture. (**A**) *In vitro* transformation reactions were performed in the presence of microsomal fractions prepared from different tissues of *Mizuhopecten yessoensis.* From the front side to the backside, the microsomal fraction was prepared from the gill (purple), gonad (green), adductor muscle (blue), digestive gland (black) and mantle (red) and was subjected to *in vitro* acylation reactions. The production of 7-*O*-palmitoyl OA was confirmed by LC-MS/MS analysis on multiple reaction monitoring (MRM) negative ion mode with *m/z* 1041 > 255 as precursor ion/product ion; (**B**) Mass chromatograms for *m/z* 1041 > 255 from sample (black) and three mass chromatograms for *m/z* 803 > 255 (green, OA), *m/z* 817 > 255 (red, DTX1) and *m/z* 1055 > 255 (blue, 7-*O*-Palmitoyl DTX1) from diarrhetic shellfish poisoning (DSP) standard were obtained from LC-MS/MS on MRM negative ion mode. The latter three chromatograms were overlapped and shown in the front side; (**C**) The MS/MS spectrum for 7-*O*-palmitoyl OA. The peak at 18.2 min in the mass chromatogram in (**A**) was analyzed with LC-MS/MS on enhanced product ion (EPI) scan mode. (**D**) The estimated structures for the three product ions *m/z* 1041, *m/z* 785 (D-3) and *m/z* 255 (D-1, D-2) observed in the MS/MS spectra in (**C**).

When OA was incubated with the microsomal fraction in the presence of palmitoyl CoA and ATP, the MRM peak clearly appeared at 18.2 min (Figure 3A). In fact, the DSP toxins standard contained OA, DTX1 and lipid adducts to DTX1 (7-*O*-acyl DTX1), but not the lipid adduct to OA (7-*O*-acyl OA), and it was necessary to confirm that the signal could be assigned to that for 7-*O*-palmitoyl OA. Due to the structural difference between OA and DTX1 at the substituent at C-35 (H or CH_3_), we assumed that the relative retention time for DTX1 to OA would be similar to that for 7-*O*-palmitoyl DTX1 to 7-*O*-palmitoyl OA. The retention times for DTX1 and OA was 11.7 min and 10.6 min, respectively (Figure 3B). Likewise, since 7-*O*-palmitoyl DTX1 was eluted at 19.5 min, the MRM peak with 1041 > 255 at 18.2 min was acceptable to 7-*O*-palmitoyl OA. To validate our methodology for identifying 7-*O*-palmitoyl OA, we performed LC-MS/MS on enhanced product ion (EPI) scan mode (Figure 3C) with 1041 *m/z* as the precursor ion. At the retention time of 18.2 min where the MRM peak with *m/z* 1041 > 255 was detected (Figure 3A), the MS/MS spectrum revealed two product ions at *m/z* 785 and *m/z* 255, which presumably accounted for the ion [OA − H_2_O − H]^−^ (D-3) due to loss of palmitic acid and OA fragment usually selected as the product ion (D-1) or [Palmitic acid − H]^−^ (D-2), respectively (Figure 3D). All of these speculations on the LC-MS/MS analysis clarified production of the 7-*O*-palmitoyl OA in the transformation reaction.

Stoichiometry of the transformation reaction was examined by quantitating the area of the MRM peak for the 7-*O*-palmitoyl OA in reference to that for 7-*O*-palmitoyl DTX1 in the standard mixture. The yield of 7-*O*-palmitoyl OA was 0.5% based on OA applied in the reaction (Figure 3A). Since the ratio of DTX3 to DTX1 in the toxic *M. yessoensis* was 10–14 [38], the observed transformation rate was extremely low. On the analysis with LC-MS/MS on MRM negative ion mode, we searched for other lipid adducts to OA that were produced by a reaction with endogenous lipid molecules in the protein extract, though none of the other adducts were identified. In order to assess production of DTX3 in other tissues in the *M. yessoensis*, the microsomal fractions prepared from the adductor muscle, gill, mantle and gonad were compared for the transformation ability of OA. The highest yield was obtained from the digestive gland, and a marginal yield was obtained from other tissues (Figure 3A). We thus speculated that optimization of the condition might be required to reproduce the naturally occurring transformation in the *M. yessoensis*.

**Figure 4:**
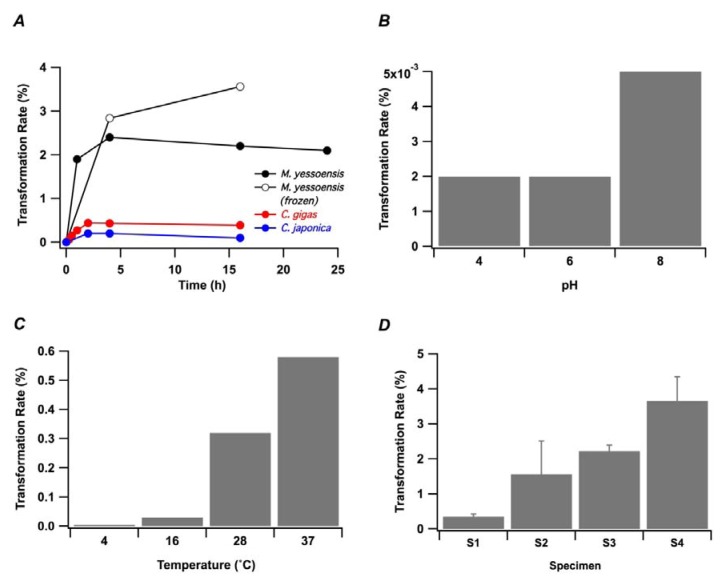
Dependence of *in vitro* transformation of OA on (**A**) shellfish species, (**B**) pH, (**C**) temperature and (**D**) specimens. (**A**) Time course of the transformation reaction at pH 6/37 °C in the presence of digestive gland extracts from *M. yessoensis* (closed circles), the frozen *M. yessoensis* (open circles), *C. gigas* (red) and *C. japonica* (blue) was monitored. (**B**–**D**) Transformation reaction was carried out at different pH/37 °C in (**B**), at pH 6/different temperature in (**C**), at pH 6/37 °C with 4 specimens in (**D**). S1, S2, S3 or S4 in (**D**) was the code name for each specimen.

A time course of the reaction was examined in Figure 4A. OA was reacted with palmitoyl CoA in the presence of a microsomal fraction of the digestive gland from live *M. yessoensis* for 15 min, 30 min, 60 min, 2 h, 4 h and 16 h. The production of 7-*O*-palmitoyl OA was increased during the initial two hours and ceased down thereafter. The transformation reaction was also observed in a similar manner when the microsomal fraction was prepared from the frozen *M. yessoensis*. 7-*O*-palmitoyl DTX1 was subjected to the same reaction condition, though it was almost 100% recovered (data not shown), suggesting that a hydrolysis of the transformation product did not occur. We then attempted to examine the effect of pH and temperature on the transformation reaction. A similar transformation rate was obtained at pH 4 and at pH 6, while it was more than 2-fold at pH 8 (Figure 4B). Also, when the transformation reactions were performed at 4 °C, 16 °C, 28 °C and 37 °C, a higher transformation rate was obtained at a higher temperature (Figure 4C).

Suzuki *et al.* first obtained direct evidence on the transformation of OA into DTX3 in 1999 [9]. Non-toxic *M. yessoensis* were fed with the toxic dinoflagellate *Dinophysis fortii*. In the digestive gland of *M. yessoensis*, most of the OA was converted to 7-*O*-palmitoyl OA. 

Rossignoli *et al.* injected agar capsules containing OA into live *Mytilus edulis* and continued injecting it for 12 days [10]. They found that the amount of OA and DTX3 (7-*O*-acyl OA) accumulated in the digestive gland of *M. edulis* gradually increased and that the lipid conjugated to 7-OH was not only palmitic acid (C16:0), but also C14:0, C15:0, C16:1, C17:0, C18:1, C18:2, C18:4, C20:5 and so on. They also demonstrated the first *in vitro* transformation of OA in the presence of the microsomal fraction of the *M. edulis*. The significance of our work was that we provided more evidence for *in vitro* transformation in the *M. yessoensis* extract with the assistance of acyl CoA transferase. Furthermore, kinetics and stoichiometry of the reaction were revealed. The transformation reaction from OA to 7-*O*-palmitoyl OA got saturated within 2 h and did not proceed even when a longer incubation period was applied. The transformation rate varied from 0.01% to 3%, and the rest of the input OA was recovered. Hydrolysis of 7-*O*-palmitoyl DTX1 to regenerate DTX1 did not occur, though the crude extract should have contained esterase, as exemplified by the hydrolysis of the diol esters of OA to OA in the digestive gland of Greenshell mussel *Perna canaliculus* [18]. The ratio of DTX3/DTX1 accumulated in naturally intoxicated *M. yessoensis* was more than 10, which explained the low efficacy of the *in vitro* transformation [38]. Since the difference between *in vivo* and *in vitro* experiments should not be ignored, the transformation could be hampered by other factors. A degradation of the enzyme or depletion of palmitoyl CoA applied to the reaction might have occurred. Otherwise, the dissociation of 7-*O*-palmitoyl OA from the enzyme could not be properly regulated in the *in vitro* system.

The LC-MS/MS analysis on an MRM negative ion mode implied substrate specificity for the OA/DTX-transforming enzyme (Figure 3B). The relative retention time of the *m/z* 1041 > 255 peak (18.2 min) to 7-*O*-palmitoyl DTX1 (19.5 min) was identical to the relative retention time of OA (10.6 min) to DTX1 (11.7 min). No other peak appeared in the *m/z* 1041 > 255 mass chromatogram, suggesting that the acylation proceeded regioselectively at C7-OH in spite of the fact that there were four hydroxyl substituents in OA.

As for another acyl-CoA transferase to the hydroxyl group, the acyl-CoA:steroid acyltransferase from the digestive gland of the oyster *Crassostrea virginica* was identified, which did not only catalyze acylation at 3β-OH, but also at 17β-OH of the steroid hormones [37], indicating that the enzyme cannot distinguish minute differences in the substrate. In contrast to the OA/DTX-transforming enzyme, the optimum pH of the acyl-CoA:steroid acyltransferase from *C. virginica* was around 6.0. In a similar fashion to the OA/DTX-transforming enzyme, the reaction yield became higher as the temperature was increased. The rat liver microsome also had enzymatic activity that promoted acylation to the hydroxyl group of retinol [39,40]. Contrary to the *in vitro* transformation reaction of OA observed in this study, the enzymatic activity turned out to be the most intense during pH 6–7. Furthermore, the transformation proceeded even in the absence of acyl-CoA, and the yield was markedly increased in the presence of acyl-CoA.

### 2.3. *In Vitro* Transformation of OA into 7-*O*-Palmitoyl OA (DTX3) in Other Bivalve Species

In order to compare the *in vitro* transformation reaction between different bivalve species, the bivalves *C. japonica* and *C. gigas* were chosen. Since a sufficient amount of protein was not obtained from the digestive gland of a single *C. japonica*, multiple specimens were treated. It turned out that the two bivalves’ extract transformed OA to 7-*O*-palmitoyl OA at the lower rate than the *M. yessoensis* extract (Figure 4A). It is important to address that the transformation reaction was observed in the extracts from three bivalve species *M. yessoensis*, *C. gigas* and *C. japonica*. The first two species were the subject of monitoring DSP toxins, while *C. japonica* has not been reported for intoxication of DSP. Without being scientifically provided, the reason why *C. japonica* have not been the vector of the diarrhetic shellfish poisoning would be speculated as follows: (1) *C. japonica* exists in brackish-water region that may not be accessible to *Dinophysis* spp.; (2) The depth range of habitat is different between the two species; and (3) The locations for the *C. japonica* industry is limited and the incidents of human intoxication could occur at much less frequency compared to the incidents derived from scallop. We then assessed the difference among specimens on transformation ability, and four *M. yessoensis* specimens were tested for the reaction. As a consequence, an 8-fold difference at maximum was observed among those specimens (Figure 4D), which made it difficult to compare the transformation efficacy among different species shown in Figure 4A. However, the enzymatic activity recognized in *C. japonica* let us propose that the transformation reaction could be a fundamental system that commonly exists in the bivalve species.

The absence of OABP2 in bivalves postulated uniqueness of the protein in the *H. okadai*. In addition, the result raised a question why the DSP toxin-containing bivalves were tolerant to DSP-toxins, since depuration of DSP toxins depends on bivalve species and usually takes 2–3 weeks [41,42]. Various transformation reactions were reported for shellfish toxins in the digestive gland of host species. Pectenotoxin, which used to be classified as DSP toxin, was synthesized by the *Dinophysis* spp. and was subjected to hydrolysis to its seco acid in the Greenshell™ mussel *Perna canaliculus* [18,43]. These transformations could be programmed in each species to reduce risk of self-intoxication.

### 2.4. Characterization of OA-Transforming Enzyme with an Attempt of the Purification

We then attempted to characterize the transformation enzyme by a simple separation technique. The microsomal fraction was sonicated in the presence of EDTA to obtain the soluble fraction. Both the supernatant and pellet were subjected to the transformation reaction. Enzymatic activity was not observed in the supernatant and remained in the pellet, suggesting that the enzyme did not become soluble under the mild condition. Other acyl transferases reported in literature did not necessarily share this intrinsic nature. The enzymatic activity contributing pregnenolone esterification, considered as a potential detoxification mechanism in *Saccharomycess cerevisiae*, was extracted to the soluble fraction without using surfactant [44]. Likewise, acyltransferase identified in *Ascaris suum* also resided in the soluble fraction [45]. On the other hand, the membrane-associated property of acyl-CoA:cholesterol *O*-acyltransferase was addressed by Spector *et al.* in 1979, where the enzyme fractions were extracted with triton X-100 [46]. Further separation of the OA/DTX-transforming enzyme would be required to address the membrane-intrinsic nature.

## 3. Experimental Section

### 3.1. Materials

Due to outbreak of the dinoflagellate *Dinophysis fortii*, shellfishing was prohibited from July 7 to July 27 in 2010 for the scallop *Mizuhopecten yessoensis* and from July 8 to September 2 in 2010 for the ascidian *Halocynthia roretzi*. Toxic specimens of *M. yessoensis*, the *Mytilus galloprovincialis* and *H. roretzi* were collected in Yamada Bay, Shimohei District, Iwate Prefecture, Japan on 26 July 2010. Live nontoxic bivalves *M. yessoensis*, *Crassostrea gigas* and *Corbicula japonica* were purchased from a fish market and transported to the laboratory on ice. The National Research Institute of Fisheries Science provided the DSP toxin standard cocktail [47]. OA, palmitic acid and palmitoyl coenzyme A were purchased from Wako Pure Chemicals. ATP was purchased from Oriental Yeast Co., Ltd. A part of the OA was purified from the sponge *H. okadai*. The purity of the OA was 73% and that of the DTX1 was 12%, which were determined by LC-MS/MS in reference to the purchased OA. [24-^3^H]OA and 7-*O*-palmitoyl DTX1 (DTX3) were kindly provided by Dr. Takeshi Yasumoto of Japan Food Research Laboratories.

### 3.2. Preparation of the Microsomal and Mitochondrial Fractions from Bivalves

The fresh specimen was dissected into five tissues of gill, gonad, adductor muscle, digestive gland, and mantle by scissors and homogenized with Ultra Turrax T25 (IKA Japan K.K., Osaka, Japan) in 4 volumes of a buffer containing 10 mM Tris/HCl (pH 7.6), 150 mM KCl, 500 mM sucrose and Protease Inhibitor Cocktail for Use with Mammalian Cell and Tissue Extracts (Nacalai Tesque, Kyoto, Japan). The crude suspension was successively centrifuged at 800× *g* for 10 min, 11,000× *g* for 15 min and 20,500× *g* for 15 min. In the first two centrifugations, the supernatant was taken for the next step. In the final centrifugation, the supernatant and pellet were stored as the microsomal and mitochondrial fractions, respectively [37]. The protein concentration of these fractions were determined with Protein Assay CBB Solution (Nacalai Tesque, Kyoto, Japan) and used for the following transformation reaction.

### 3.3. *In Vitro* Transformation Reaction

1.0 μg (1.2 nmol) of OA, 26 μg (26 nmol) of palmitoyl coenzyme A and 1.3 mg (2.6 μmol) of ATP were incubated with 2.0 mg of the microsomal fraction. The reaction mixture was extracted three times with ethyl acetate (1 mL). The organic phase was concentrated under a flow of nitrogen gas and was dissolved in methanol. LC-MS/MS analysis was carried out as described in literature. Briefly, the LC-MS/MS analysis on MRM negative ion mode on acyl OA analogues was performed using the following ion channels, with a dwell time of 22 ms, a declustering potential of −150 V, an entrance potential of −10.5 V and a collision energy of −84 V for each analogue; 14:0-OA; 1013 > 227, 15:0-OA; 1027 > 241, 16:0-OA; 1041 > 255, 17:0-OA; 1055 > 269, 18:0-OA; 1069 > 283, 20:0-OA; 1097 > 311, 22:0-OA; 1125 > 339, 14:1-OA; 1011 > 225, 16:1-OA; 1039 > 253, 18:1-OA; 1067 > 281, 20:1-OA; 1095 > 309, 18:2-OA; 1065 > 279, 18:3-OA; 1063 > 277, 18:4-OA; 1061 > 275, 20:4-OA; 1089 > 303, 18:5-OA; 1059 > 273, 20:5-OA; 1087 > 301, 22:6-OA; 1113 > 327. The toxin calibrants of OA, DTX1 and DTX3 (7-*O*-palmitoyl-OA) from toxin standards distribution project in Japan were used [47].

### 3.4. Binding Assay

2.5 nM of [24-^3^H]OA was incubated on ice for 2 h with 5.0 μg of protein extract obtained from the bivalve species in 100 μL of a binding buffer consisting of 20 mM Tris/HCl, 150 mM NaCl, 1.0 mM EDTA and 0.01% Tween 20 (pH 7.4). The experiments were performed in the absence and presence of 100 nM of non-radioactive OA to estimate nonspecific binding of the ligand. The reaction mixture was directly added to the Nick column (GE Health Care Japan, Tokyo, Japan), and the protein fraction was collected with 750 μL of the binding buffer. The radioactivity was measured with a liquid scintillation counter to determine the specific binding of [24-^3^H]OA.

## 4. Conclusions

The absence of OABP2 in shellfish emphasized uniqueness of the protein in the *H. okadai*. The acylation of OA into DTX3 only underwent in the presence of the digestive gland extract of shellfish under assistance of palmitoyl CoA and proceeded regioselectively. We postulated that the acylation was a fundamental mechanism for the shellfish.
